# Increased amounts of cell-free DNA released from a culture with a high content of cancer stem cells

**DOI:** 10.3389/fcell.2025.1499936

**Published:** 2025-03-28

**Authors:** Ileana J. Fernández-Domínguez, Enrique Pérez-Cárdenas, Lucia Taja-Chayeb, Talia Wegman-Ostrosky, Claudia H. S. Caro-Sánchez, Alejandro Zentella-Dehesa, Alfonso Dueñas-González, Horacio López-Basabe, Rocío Morales-Bárcenas, Catalina Trejo-Becerril

**Affiliations:** ^1^ Subdirección de Investigación Básica, Instituto Nacional de Cancerología, México City, Mexico; ^2^ Posgrado en Ciencias Biológicas, Universidad Nacional Autónoma de México. Edificio D, 1° Piso, Circuito de Posgrados, Ciudad Universitaria, México City, Mexico; ^3^ Departamento de Patología del Instituto Nacional de Cancerología, México City, Mexico; ^4^ Departamento de Medicina Genómica y Toxicología Ambiental, Instituto de Investigaciones Biomédicas (IIBO), Universidad Nacional Autónoma de México (UNAM), México City, Mexico; ^5^ Unidad de Bioquímica, Instituto Nacional de Ciencias Médicas y Nutrición Salvador Zubirán (INCMNSZ), México City, Mexico; ^6^ Departamento de Gastroenterología del Instituto Nacional de Cancerología, México City, Mexico

**Keywords:** cell-free DNA, horizontal DNA transfer, cancer stem cells, colorectal cancer, cellular transformation, 3D culture

## Abstract

**Background:**

The study and characterization of cell-free DNA (cfDNA) has gained significant importance due to its clinical applications as a diagnostic and prognostic marker. However, it remains unclear whether all cell populations within a tumor or culture contribute equally to its release. This pioneering research analyzes the contribution of cancer stem cells (CSCs) in colon cancer cell lines to the amount of cfDNA released and its role in cellular transformation.

**Methods:**

The CSC population derived from the SW480 colon cancer cell line was enriched using a non-adhesive culture system to assess the quantity and electrophoretic profile of the released cfDNA. Subsequently, *in vitro* transformation assays were conducted to compare the transforming capacity of the cfDNA obtained from enriched cultures with that from non-enriched cultures. Group differences were analyzed using analysis of variance (ANOVA), followed by *post hoc* interpretation with Tukey’s test.

**Results:**

Our study revealed that cultures with CSCs released greater amounts of cfDNA, displaying a distinct fragment profile. Additionally, cfDNA from different cellular origins influenced the transformation characteristics of NIH3T3 cells. This is the first demonstration of a link between CSC proportions and cfDNA release, suggesting that CSCs and microenvironmental conditions can affect cfDNA quantity and its potential to induce transformation.

**Conclusion:**

These findings highlight the importance of cfDNA in carcinogenesis and its potential as a biomarker and therapeutic target, especially given the role of CSCs in drug resistance and tumor aggressiveness.

## 1 Introduction

Cancer is one of the most complex pathologies and difficult to eradicate, making it a leading cause of death worldwide. Colorectal cancer (CRC) is the third most frequently diagnosed malignancy and the second leading cause of cancer-related mortality (Sung et al., 2021). Given these statistics, there is a significant need for diagnostic methods capable of detecting the disease at an early stage. Recent studies have explored markers as potential diagnostic tools for colorectal cancer (CRC) (Łukaszewicz-Zając and Mroczko, 2021). In particular, cell-free DNA (cfDNA) has been demonstrated to provide genetic and epigenetic information from primary and metastatic CRC, facilitating the assessment of tumor heterogeneity (Xie and Kim, 2021).

cfDNA is found in various body fluids, such as blood, saliva, urine, cerebrospinal fluid, lymphatic fluid, bile, and milk, among others (Dasari et al., 2020; Fernández-Domínguez et al., 2021). It is also present in the supernatant of cultures of different cell lines (Bronkhorst et al., 2021). cfDNA has multiple origins and can be released from different mechanisms (Bi et al., 2020), resulting in several molecular structures in the extracellular environment, including apoptotic bodies, exosomes, microvesicles, nucleosomes, and macromolecular complexes linked to lipids, proteins, or RNA (Tutanov and Tamkovich, 2022).

cfDNA can have varied sizes depending on whether its release is direct or indirect. Direct release involves a metabolically active secretion from living cells, where fragments of 1,000 to 3,000 bp are associated with DNA-protein binding and larger fragments are related to extrachromosomal circular DNA and extracellular vesicles (30–20,000 bp) (Aucamp et al., 2018). Conversely, the indirect form of release is a consequence of different cell death types, such as apoptosis and necrosis, which produce fragments ranging in size from 147 to 200 bp (apoptotic cells) and approximately 10,000 bp (necrotic cells) (Fernández-Domínguez et al., 2021; Aucamp et al., 2018).

The diversity of fragment sizes and the genetic and epigenetic variants of cfDNA are derived from tumor heterogeneity (Bronkhorst et al., 2022), a product of the different cell populations constituting the tumor burden. Cancer stem cells (CSCs) are a critical cellular subpopulation involved in metastasis, tumor recurrence, and resistance to oncological treatments. These cells exhibit distinctive features, including the ability to dedifferentiate, self-renew, express high levels of anti-apoptotic genes, and utilize aberrant DNA repair mechanisms (Hatano et al., 2017; Liu K et al., 2022; Mancini et al., 2018). CSC are predominantly located in the G1 phase of the cell cycle but can transition into a quiescent state (Liu K et al., 2022). They are considered key initiators of carcinogenesis due to their ability to differentiate into various cell lineages (De Francesco et al., 2018), contributing to tumor heterogeneity and plasticity. This capacity facilitates tumor reconstitution, growth, maintenance, and progression (Liu X et al., 2022; Wahab et al., 2017). Despite their high clinical significance, CSCs represent only a small proportion of the tumor mass, ranging from 0.01% to 2% (Liu X et al., 2022).

Their isolation and characterization rely on methodologies employing antibodies targeting specific cell surface markers predominantly expressed in CSCs, such as CD133+, CD44^+^, and CD24^+^, along with the enzyme ALDH and ABC transporters. However, the expression of these markers can vary among tumors and cell lines, fostering both intra- and intertumoral heterogeneity (Hatano et al., 2017).

In colon cancer, cancer stem cells (CCSCs) represent a heterogeneous subpopulation with varying levels of differentiation (Pan et al., 2017). They may arise from normal colon stem cells undergoing a malignant transformation driven by the tumor microenvironment or dedifferentiated cancer cells (Pan et al., 2017). CCSCs influence the surrounding cellular environment through cell-to-cell interactions or paracrine signaling, affecting the behavior of neighboring cells (Liu X et al., 2022). Additionally, *in vitro* sphere-forming assays using low-adhesion 3D colon cancer cultures allow for the identification and enrichment of CCSCs (Ayob and Ramasamy, 2018; Pan et al., 2017).

Recent studies have explored the cellular processes involved in cfDNA generation, revealing that cfDNA levels and fragmentation profiles vary depending on sample origin, cell lineage, culture conditions, metabolic activity, and growth phase (Bronkhorst et al., 2016; Aucamp et al., 2017a; Ungerer et al., 2022). Furthermore, differences in extraction, quantification, and processing methods significantly influence the results obtained (Bronkhorst et al., 2022).

To date, all published studies have derived cfDNA from the total population, which represents the entirety of the tumor and includes cfDNA released by various cell types and tissues, resulting in heterogeneous cfDNA populations (Ungerer et al., 2022; Sanchez et al., 2021; Liu Y, 2022; Cristiano et al., 2019). However, the individual contributions of different cellular populations within the tumor mass to cfDNA, particularly those from the subset of CSCs, remain poorly understood. CSCs are increasingly recognized as a critical cellular population in tumorigenesis.

One of the few relevant studies is by Liu ZB et al. (2019), who identified specific mutations in CSCs (MLF2 R158W, RPL39 A14V, HN1L P20L, and HN1L A106V) within the cfDNA of breast cancer patients. This study demonstrated that detecting CSC-associated mutations in cfDNA is feasible and serves as a highly sensitive biomarker of tumor burden. Notably, the fractional abundance of these mutations was shown to increase from early to advanced stages of the disease and in metastases. Nevertheless, no research has explored whether there is a correlation between the number of CSCs and the amount of cfDNA released.

This work aimed to evaluate whether the proportion of CSCs influences the amount of cfDNA released. We used an *in vitro model* of colon cancer cell lines with varying proportions of CSCs. We then evaluated the amount of cfDNA released and its electrophoretic profile. Additionally, we performed *in vitro* cell transformation assays with the obtained cfDNA.

## 2 Materials and methods

### 2.1 Cell lines and culture

Colon cancer cell lines (SW480, HCT116, and HT29) and the mouse embryonic fibroblast cell line (NIH3T3) were maintained in the DMEM-F12 medium (Gibco). The pluripotent embryonal carcinoma cell line (NCCIT) was cultured in RPMI-1640 medium (Gibco). All cultures were supplemented with 10% FBS (fetal bovine serum) (Gibco), 100 U/mL penicillin (Gibco), and 0.1 mg/mL streptomycin (Gibco), and incubated at 37°C in a humidified atmosphere with 5% CO_2_. All cell lines were purchased from the American Type Culture Collection (ATCC).

### 2.2 Non-adhesive culture

To enrich the cellular population with CSC characteristics (sphere formation and the expression of stemness-associated markers), we utilized the modified non-adhesive culture system described by Chen et al. (2012). Briefly, adherent parental SW480 cells (A-SW480) were dissociated with trypsin into a single-cell suspension and seeded at a density of 2 × 10^4^ live cells per well in 6-well plates. These plates were coated with a thin film of 1.2% agarose (Invitrogen™) dissolved in sterile water. The culture medium (DMEM-F12) was changed every other day until sphere formation. The non-adherent cells obtained from this culture system were designated S-SW480 (spheres SW480). The sphere cultures were evaluated at three different time points: 3, 5, and 9 days, defined as S3D, S5D, and S9D, respectively. Their adherent counterparts were named A3D, A5D, and A9D.

### 2.3 Supernatant preparation

The colon cancer cell lines were cultivated in 75 cm^2^ cell culture flasks under the specified conditions. The medium was discarded upon reaching 70%–80% confluence, and the cells were washed twice with sterile PBS buffer. The cell cultures were then incubated with a medium containing 2% FBS for 48 h at 37°C and 5% CO_2_. Following this incubation period, the supernatants (conditioned medium) were transferred into 50 mL conical centrifuge tubes and cleared of any residual cells and debris by centrifugation at 400 xg for 20 min. The supernatants were passed through a 0.45 µm filter to eliminate any remaining cells. Aliquots of each supernatant sample were seeded into 25 cm^2^ culture flasks and incubated at 37°C for 1 week to verify the absence of cellular growth. For cfDNA analysis, 120 mL of supernatant was concentrated to 12 mL using an ultrafiltration system with a 10 kDa pore-size membrane. The concentrated supernatant was then immediately processed for cfDNA extraction.

### 2.4 Extraction and quantification of cfDNA

cfDNA extraction was performed directly from the concentrated supernatant using SDS/proteinase K digestion followed by phenol/chloroform extraction as described by Anker et al. (1994). Briefly, 500 µL of supernatant was mixed with 500 µL of a solution of SDS/proteinase K (20 mg/mL) (Invitrogen) and incubated overnight at 55°C. An equal volume of phenol/chloroform (1∶1 v/v) was added, gently mixed by inversion for 10 min, and centrifuged at 9,600 xg for 10 min. The aqueous phase was recovered and mixed with an equal volume of chloroform, gently mixed by inversion for 10 min until an emulsion formed, and centrifuged at 9,600 g for 10 min. The upper aqueous phase was precipitated overnight at −20°C with 1/10 volume of 7.5 M ammonium acetate, 1 µL of glycogen, and 2.5 volumes of isopropyl alcohol, then centrifuged at 18,000 g for 45 min at 4°C. The cfDNA pellet was washed once with 70% ethanol, air-dried, and solubilized in warm water. Total cfDNA was quantified using the *PicoGreen* assay (Invitrogen™) following the manufacturer’s instructions. cfDNA was measured using a fluorometer.

### 2.5 Genomic DNA extraction

Genomic DNA was extracted using phenol, chloroform, and ethanol. DNA samples were then resuspended in nuclease-free water, quantified spectrophotometrically, and stored at −20°C.

### 2.6 Cell cycle analysis

Cell cycle analysis was conducted using propidium iodide staining. After 48 h of culturing of A9D and S9D cells with DMEM-F12 containing 2% of FBS, the cells were washed twice in PBS and detached by mechanical dissociation in PBS containing 1 mM EDTA. Next, cells were collected and centrifuged at 340 xg for 5 min. The resulting pellet was resuspended and fixed in 70% ethanol at −20°C. The cells were then incubated with 100 µL of trypsin for 10 min at room temperature. Subsequently, 100 µL of RNase A combined with trypsin inhibitor was added for a second 10 min incubation. Finally, the cells were incubated with 80 µL of propidium iodide for 15 min in the dark at 4°C. Samples were evaluated by flow cytometry (BD FACSCanto™ II), and the data obtained were analyzed using ModFit LT™ software.

### 2.7 Proliferation assay

Proliferation was measured based on the expression of the specific marker Ki-67. Briefly, cells were harvested, washed once with cold PBS, resuspended in 500 µL of fixation solution (4% formaldehyde), and incubated for 10 min at room temperature. Following this incubation, 2 mL of cold PBS was added, and the cells were centrifuged for 5 min at 340 xg to remove the fixative solution. Subsequently, the cells were resuspended and incubated for 15 min in 500 µL of permeabilization solution (PBS-0.1% Triton X-100) at room temperature. They were washed with cold PBS and blocked with PBS-10% goat serum for 30 min. Cells were washed with PBS and stained with diluted Ki-67 antibody (sc-23900, Santa Cruz Biotechnology) in 100 µL PBS with 1% FBS for 30 min at room temperature in the dark. The cells were analyzed using flow cytometry.

### 2.8 CD133^+^ and CD44^+^ expression analysis

The expression profiles of CD133^+^ and CD44^+^ were analyzed by flow cytometry. Briefly, cells were trypsinized into single-cell suspension and washed twice with cold PBS. Subsequently, 5 × 10^5^ cells/mL were incubated with 100 µL of 3% FBS in PBS containing PE-conjugated anti-CD133 (566,593, BD Pharmingen™) and APC-conjugated anti-CD44 (ab81424, Abcam) for 45 min in the dark at 4°C. After incubation, the unbound antibodies were washed off by adding 1 mL of cold PBS and centrifuging at 340 x g for 5 min. Cells were resuspended in 500 µL of PBS with 1% FBS and analyzed by flow cytometer. Isotype PE-IgG1κ (554,680, BD Pharmingen™), APC-IgG2bκ (555,745, BD Pharmingen™), and unstained cells served as negative controls.

### 2.9 Extraction and quantification of cellular proteins

Cells were washed with cold PBS, centrifuged at 340 xg for 5 min, and resuspended in ice-cold RIPA lysis buffer (Thermo Fisher) supplemented with protease and phosphatase inhibitor cocktail (Thermo Fisher). The cells were then disrupted by sonication. After five sonication cycles at 50% for 5 s each (with samples kept on ice between cycles), the samples were centrifuged at 18,000 g for 15 min at 4°C to remove cellular debris. The supernatant was designated as whole cell lysate. The total cellular protein content was determined using the Bradford method.

### 2.10 Western blot analysis

For immunoblot analysis, whole cell lysates (30 µg of protein) were separated by electrophoresis on 8%–10% SDS-PAGE and electrotransferred to PVDF membranes using a wet blotting system. Membranes were blocked with 5% low-fat milk in TBS-0.1%TWEEN®20 under agitation for 1 h at room temperature. The antibodies tested included Oct-3/4 (1∶1,000; sc-365509, Santa Cruz Biotechnology), Nanog (1∶1,000; sc-374001, Santa Cruz Biotechnology), Sox2 (1∶1,000; sc-365964, Santa Cruz Biotechnology), E-cadherin (1:1,000; sc-8426, Santa Cruz Biotechnology), N-cadherin (1:1,000; sc-59987, Santa Cruz Biotechnology), and Vimentin (1∶1,000; sc-32322, Santa Cruz Biotechnology), which were incubated in TBS-0.1%Tween 20 buffer containing 1% BSA at 4°C overnight under constant agitation. β-Actin was used as a housekeeping protein in a dilution of 1:2,500 (sc-8432, Santa Cruz Biotechnology). After incubation, membranes were washed with TBS-0.1% TWEEN®20 and incubated with a secondary antibody m-IgGκ BP-HRP (1:10,000; sc-516102, Santa Cruz Biotechnology) in TBS-0.1% TWEEN®20 buffer containing 5% low-fat milk for 1 h at room temperature. The immunodetection was performed with chemiluminescence peroxidase substrate (34,577, Thermo Fisher), and the luminescence was visualized on X-ray film. A densitometry analysis was performed using the ImageJ software (NIH, Bethesda, Maryland, United States).

### 2.11 Polyacrylamide gel electrophoresis (PAGE)

The integrity and fragment profile of the cfDNA was analyzed using PAGE. Briefly, polyacrylamide gels were prepared at different concentrations (6% and 12%) and run with 1X TBE buffer (Sigma-Merck) for 2 h at 80 V. The 6% gels were used to evaluate the integrity and quantity of cfDNA released into the extracellular medium. In comparison, the 12% gels were used to achieve adequate separation of the different sizes of cfDNA fragments. A total mass of 250 ng of cfDNA was loaded into each well to demonstrate the profile corresponding to each supernatant. The gels were stained with SYBR™ Gold nucleic acid gel stain (Invitrogen™) for 20 min in the dark with constant agitation at room temperature. The profile of the fragments was visualized with Gel Documentation System.

### 2.12 Characterization of cfDNA fragment size

The cfDNA fragment size was analyzed using capillary electrophoresis (CE). Briefly, cfDNA was extracted as previously described and placed on a high-sensitivity DNA microfluidic chip (Agilent) according to the manufacturer’s instructions, using the Agilent 2,100 Bioanalyzer equipped with Expert 2,100 software. The results are displayed as electropherograms, showing two significant peaks at 35 bp and 10,380 bp. These peaks correspond to the size markers used to calculate the cfDNA size.

### 2.13 Global DNA methylation assay

Genomic DNA and cfDNA were isolated as described above. To detect epigenetic markers, the MethylFlash™ Global DNA Methylation (5-mC) ELISA Easy Kit (Epigentek) was used according to the manufacturer’s instructions. Briefly, 125 ng of DNA (genomic or cell-free) was added in each well of a 96-well plate and probed with an anti-5-methylcytosine antibody and HRP-linked secondary to produce a colorimetric reaction. The plate was incubated at 37°C for 60 min. Colorimetric quantification was performed using a microplate reader (Thermo Fisher) at an absorbance of 450 nm. The percentage methylation was calculated using the following equation supplied by the manufacturer: 5−mC%= (Sample OD−Negative Control)/(Slope of standard curve × S) × 100%, where S is the amount of input sample DNA in ng.

### 2.14 Passive transfection assay

NIH3T3 cells, which were used as recipients for the transformation assays, were seeded in 24-well plates and exposed to supernatants of A9D and S9D in a 1:3 ratio (fresh DMEM-F12 with 2% FBS/supernatant) for 21 days refreshing the medium every 24 h (Anker et al., 1994). After 21 days of exposure, the cells were dispersed and propagated under standard conditions. The exposed cells were then analyzed by PCR for the presence of mutated human *KRAS* sequences. Experiments were performed in biological sextuplicate and technical duplicates.

### 2.15 Transformation assays

The NIH3T3 cells obtained from the passive transfection assays were named NIH3T3-A9D and NIH3T3-S9D, respectively. We analyzed the characteristics used as indicators of malignant transformation: morphological changes, loss of contact inhibition (focus formation), and anchorage-independent growth (growth in soft agar) (Raptis and Vultur, 2001).

### 2.16 Morphological analysis

NIH3T3-A9D and NIH3T3-S9D were examined for foci formation and counted under phase-contrast microscopy.

### 2.17 Colony formation assay

A colony formation assay was performed in soft agar. Briefly, foci were isolated from plastic culture plates and expanded for analysis in soft agar. After trypsinization, cells were suspended in a DMEM-F12 medium containing 0.3% agar and 15% FBS. This suspension was plated on a medium layer containing 0.7% agar without serum. Cells were plated at a density of 6 × 10^4^ cells per 10-cm dish. After 14 days of culture, the number of colonies was scored using inverted phase-contrast microscopy. A colony was defined as containing at least 50 cells.

### 2.18 PCR

To confirm the presence of human DNA (derived from SW480) following the transfection assays, DNA was isolated from NIH3T3, NIH3T3-A9D, and NIH3T3-S9D cells. Subsequently, the human *KRAS* gene fragments and the *ALU Yd6* human sequences were amplified. Each reaction was performed in a 20 µL volume containing 100 ng of template DNA, 10 mmol/L Tris-HCl (pH 8.3), 40 mmol/L KCl, 2 mmol/L MgCl_2_ for *KRAS* (5 mmol/L MgCl_2_ for *ALU Yd6*), 200 μmol/L of each dNTP, 0.25 U Taq polymerase (Invitrogen™), and 1 μmol/L of each specific primer. The primer sequences used were as follows: Human *KRAS*: 5′-GACTGAATATAAACTTGTGGTAGT-3′ and 5′-GGACGAATATGATCCAACAATAG-3' (107 bp amplicon) and *ALU* Yd6: 5′-GAGATCGAGACCACGGTGAAA-3′ and 5′-TTGCTCTGAGGCAGAGTTT-3' (200 bp amplicon) (Walker et al., 2003). The PCR protocol included an initial denaturation step at 94°C for 5 min, followed by 40 amplification cycles, and a final extension at 72°C for 5 min. Each cycle consisted of denaturation at 94°C for 30 s, annealing for 30 s (59°C for *KRAS* and 61°C for *ALU* Yd6), and extension at 72°C for 30 s. The reactions were conducted in a MiniAmp™ Plus Thermal Cycler (Applied Biosystems™). Amplification products were confirmed by agarose gel electrophoresis.

### 2.19 Statistical analysis

All results are presented as the mean ± standard deviation (SD) derived from data collected in at least three independent experiments conducted in triplicate to sextuplicate. Differences between groups were analysed using analysis of variance (ANOVA) with SigmaStat 2.03 or InerStat-A 1.3 software, followed by a *post hoc* Tukey test for interpretation. P-values less than or equal to 0.05 were considered statistically significant.

## 3 Results

### 3.1 Cell lines with a higher proportion of CSCs release a greater amount of cfDNA

We selected colorectal cancer cell lines derived from patients at different stages of the disease: HT29, HCT116, and SW480. Cells were cultured under standard conditions. The supernatants of each line were collected and processed, and cfDNA and genomic DNA were isolated and quantified as described in the methodology. The results are reported per million cells.

**FIGURE 1:**
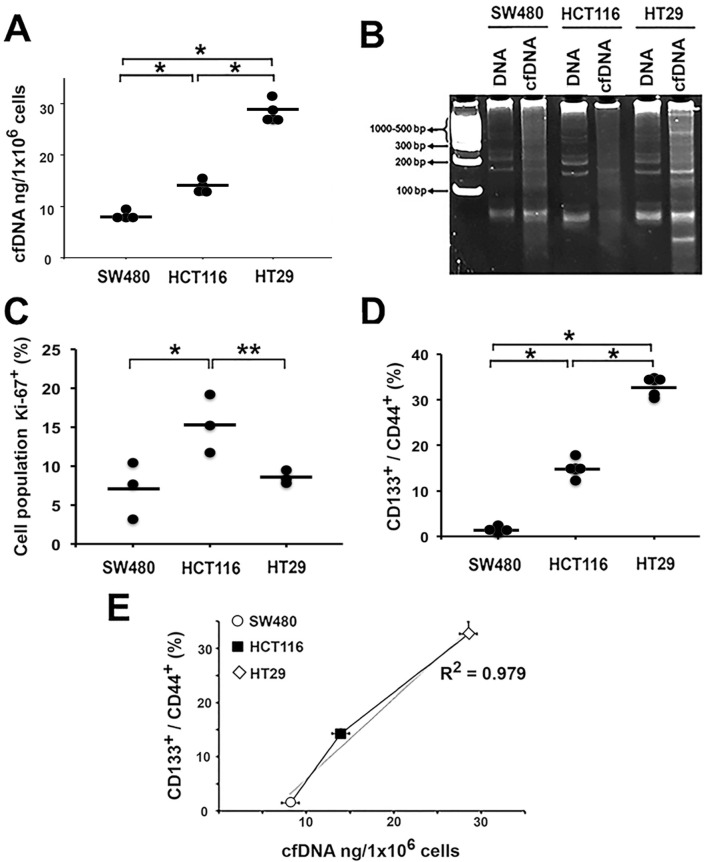
High CSC population content is associated with increased cell-free DNA (cfDNA) release. cfDNA was directly isolated from the cell culture supernatants of the colon cancer cell lines: **(A)** Quantification of cfDNA release by 1 × 10^6^ cells of each colon cancer cell line. **(B)** Determination of cfDNA fragmentation patterns by 12% PAGE: DNA = genomic DNA; cfDNA = cell-free DNA (250 ng DNA mass). **(C)** Evaluation of Ki-67 proliferation marker expression. **(D)** Expression of CD133^+^ and CD44^+^ surface markers associated with stem phenotype. **(E)** Graph the correlation between CD133^+^/CD44^+^ expression and DNA release to the extracellular medium. ^*^p < 0.05, ^**^p < 0.03.

HT29 released the highest concentration of cfDNA, followed by HCT116 and SW480, with the amounts being 29 ± 2.2 ng, 14 ± 0.9 ng, and 8 ± 0.6 ng, respectively (p < 0.01; Figure 1A). Subsequently, we analyzed the electrophoretic profile of the purified cDNAs using a 12% polyacrylamide gel (Figure 1B). The electrophoretic pattern of genomic DNA was similar in all 3 cell lines. However, for cfDNA, we observed that the DNA was more fragmented, and the electrophoretic profile differed for each line.

Fragment sizes ranged from less than 100 bp to greater than 1,000 bp, with high molecular weight fragments similar to genomic DNA. Cell viability was measured and found to be greater than 91% for all 3 cell lines (91.3% ± 1.7, 92.7% ± 2.2, and 91.8% ± 2.3 for HT29, HCT116, and SW480, respectively), suggesting that cell death contributed minimally to cfDNA release.

Additionally, we evaluated the proliferation rate of each cell line by measuring the expression of the proliferation-specific marker Ki-67. HCT116 exhibited the highest proliferation index. Interestingly, proliferation was not associated with the amount of cfDNA released. HT29, which released significantly more cfDNA, expressed Ki-67 levels comparable to those of the SW480 line, which released the least cfDNA (p < 0.05; Figure 1C).

Since we found that neither the level of cell death nor the proliferation rate could explain the differences in the amount of cfDNA released, we analyzed the cell cycle status using flow cytometry with propidium iodide staining. There were no significant differences in the G0/G1 phase of the cycle among the evaluated cell lines; however, there were differences in the S (p < 0.01) and G2/M (p < 0.05) phases. HT29 and SW480 showed a higher proportion of cells in the S phase than the HCT116 line, whereas HCT116 had a higher number of cells in the G2/M phase than SW480 and HT29 (Supplementary Figure S1).

To further investigate the origin of the differences in the amount of cfDNA released by each cell line, we analyzed whether there was a relationship between the number of stem cells and the amount of cfDNA released. We evaluated the expression of stem cell-associated surface markers CD133^+^ and CD44^+^. We found that the HT29 cell line had the highest expression of the surface markers CD133^+^/CD44^+^ (32.68% ± 0.022), while SW480 had the lowest expression (1.45% ± 0.375) (p < 0.01; Figure 1D). These findings correlate with the levels of cfDNA released, suggesting that the presence of CSCs influences cfDNA release (Figure 1E).

### 3.2 Enrichment and characterization of the CSCs population

To demonstrate that the population of CSCs was implicated in the increased amount of cfDNA released, we selected SW480, the cell line with the lowest expression of CD133^+^/CD44^+^ stemness markers (1.45% ± 0.375; Figure 1D), to enrich the population of CSCs. We utilized the modified non-adhesive culture system described previously by Chen et al. (Chen et al., 2012), which allows for selecting cells with CSC-like characteristics, such as the formation of tumor spheres (3D cell culture).

**FIGURE 2:**
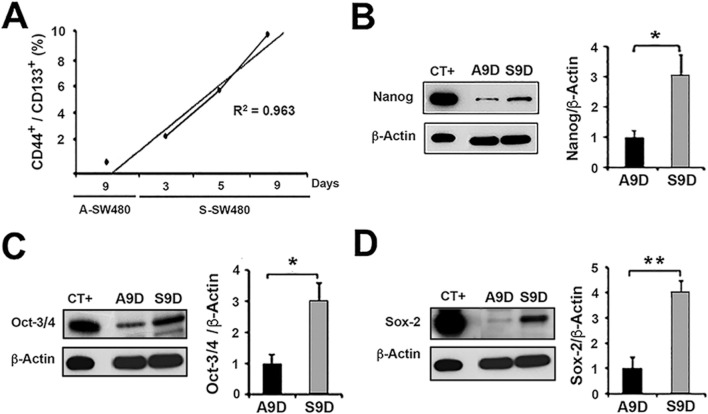
Characterization of the CSC population obtained from non-adhesive culture of cell line SW480. Non-adhesive enrichment cultures (spheres) were carried out for 3, 5, and 9 days. We observed a time-dependent enrichment and selected the non-adherent 9-day culture for further analysis (S9D). The 9-day adherent (parental) culture condition of SW480 (A9D) was used as a control. **(A)** Flow cytometry analysis of the expression of surface markers CD133^+^ and CD44^+^ in 3, 5, and 9-day non-adhesive cultures of SW480 (S-SW480) and in the adhesive culture condition (A9D). Immunoblot analysis of the expression of the transcription factors Nanog **(B)** Oct-3/4 **(C)** and Sox-2 **(D)** in A9D and S9D. CT+: Positive control. *p < 0.03, **p < 0.006.

Once the CSC-enriched culture was obtained (Supplementary Figure S2A), the expression of markers in these cultures was determined over time. We found that the co-expression of CD133^+^/CD44^+^ was time-dependent, with the highest level (9.87% ± 0.015%) on the ninth day of the culture of SW480 sphere cells (S9D) compared to parental SW480 adherent cells (A9D), which had only 0.6% ± 0.004% of co-expressing cells (Figure 2A).

To validate the enrichment of the CSC population, the transcription factors Nanog, Oct-3/4, and Sox-2, which are essential for stem cells, were evaluated by Western blot. The protein expression of these factors was higher in S9D compared to A9D (p < 0.03; p < 0.03; p < 0.006 respectively; Figures 2B–D). NCCIT, a pluripotent embryonal carcinoma cell line, was used as a positive expression control for all three proteins (CT+).

In addition to the transcription factors, the expression of E-cadherin, N-cadherin, and Vimentin, which are associated with cell differentiation, was also evaluated. N-cadherin expression (Supplementary Figure S2B) was not detected at any of the culture times (3, 5, and 9 days) evaluated in both spheres (S3D, S5D, and S9D) and adherent cells (A3D, A5D, and A9D). In contrast, E-cadherin expression (Supplementary Figure S2C) was unexpectedly higher in spheres (S9D) than in adherent cells (A9D) (p = 0.007). Regarding Vimentin expression (Supplementary Figure S2D), no differences were observed between S9D and A9D. The evaluation of these proteins was carried out in conjunction with the NCCIT cell line that served as a positive control.

### 3.3 cfDNA cell cultures enriched with CSC population release more cfDNA with different fragmentomic profiles

**FIGURE 3:**
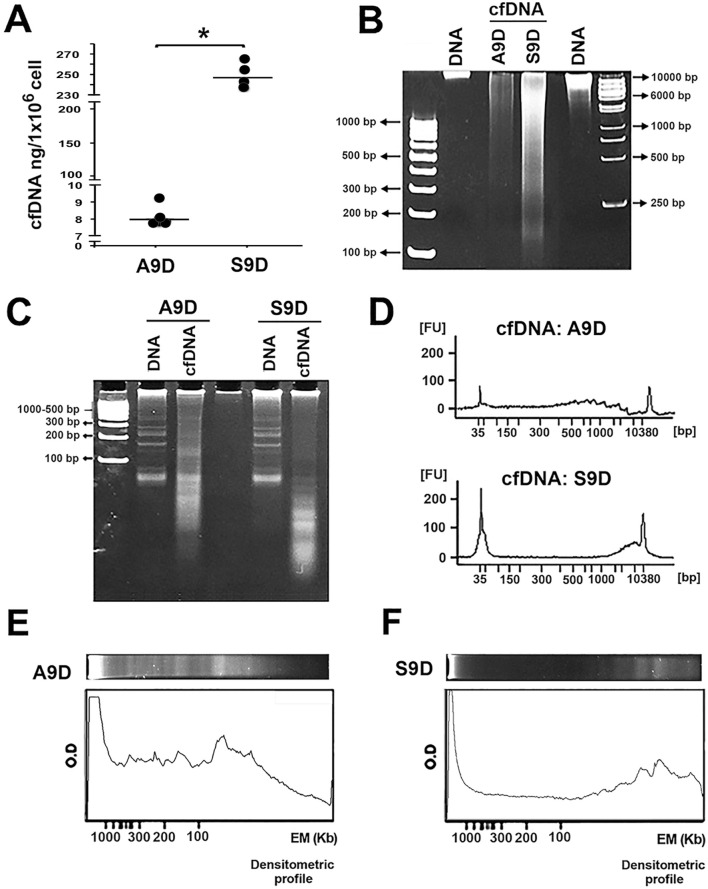
Characterization of the cfDNA released by the stem cell-enriched cell line. cfDNA was directly isolated from cell culture supernatants of S9D and A9D: **(A)** Quantification of cfDNA. **(B)** cfDNA integrity on a 6% PAGE, the amount of the electrophoresed cfDNA corresponds to that released by 1 × 10^8^ cells: DNA = Genomic DNA. **(C)** Fragmentation patterns of 250 ng of genomic DNA and cfDNA from A9D and S9D in 12% PAGE. **(D)** Capillary electropherograms of cfDNA, showing the size of the cfDNA fragments. **(E, F)** Densitometric analysis of the fragment profile obtained by PAGE shows a difference between the sizes of the fragments obtained in each culture. ^*^p < 0.001.

Once the SW480 cell culture enriched with the CSC population was obtained and characterized, we compared the amount of cfDNA released by the parental A9D and CSC-enriched S9D cells. As shown in Figure 3A, the enrichment of CSCs led to a marked increase in the amount of cfDNA released per million cells in S9D: 247 ± 12.8 ng, compared to A9D: 8 ± 0.6 ng, representing an increase of at least 30-fold, which was statistically significant (p < 0.001). To corroborate the difference in the amounts of cfDNA released by the same cell number (1 × 10^8^) in the different cultures, PAGE (6%) was performed. Figure 3B clearly shows the difference in the amount of cfDNA released in the supernatants of the A9D and S9D.

Next, we decided to analyze the electrophoretic profile of the cfDNA released by both cultures; electrophoretic profiling was performed on a 12% gel to allow better separation and visualization of the cfDNA fragments. As shown in Figure 3C, the profile of the cfDNA released by the A9D shows a continuously fragmented population ranging from a high molecular weight above 10,000 bp to a molecular weight below 100 bp. Conversely, the profile of the cfDNA released from the S9D shows two distinct fragmented populations: one with a high molecular weight above 10,000 bp and the other with a very low molecular weight below 100 bp (Figure 3C).

To better characterize these findings, we performed capillary electrophoresis. Figure 3D shows that the cfDNA released by A9D has a continuous distribution of fragments, similar, but with higher resolution, to that observed in the polyacrylamide gel. In contrast, the profile of the cfDNA released by S9D clearly shows two populations of cfDNA fragments with a discontinuous distribution, one with a high molecular weight and the other with a very low molecular weight. A densitometric analysis of the polyacrylamide gel corroborated the findings of capillary electrophoresis (Figures 3E, F).

Subsequently, global DNA methylation was determined to characterize the cfDNA released by A9D and S9D (Supplementary Figure S3). This assay aimed to evaluate whether there was any alteration in the epigenetic pattern, which would provide additional information about the heterogeneous population obtained in the supernatants. We found a slight decrease in methylation levels of cfDNA obtained from the S9D supernatant compared to that released by A9D cells; however, this difference was not statistically significant (Supplementary Figure S3). Genomic DNA methylation levels derived from both cell types were similar.

**FIGURE 4:**
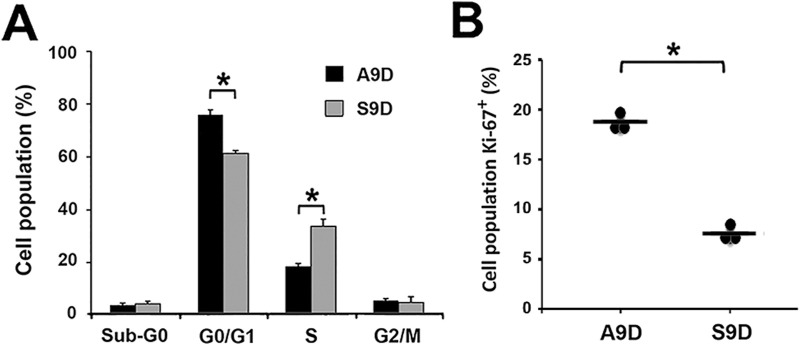
The increase in cfDNA amounts is independent of cell proliferation rate. **(A)** Cell-cycle analysis of A9D and S9D. **(B)** Expression of the proliferation marker Ki-67 in A9D and S9D cells. ^*^p < 0.01.

To explain the increased amounts of cfDNA found in the S9D supernatant, we evaluated the cell cycle status (Figure 4A). We found a significant difference in the G0/G1 and S phases between both types of cells (p < 0.01), with S9D showing a decrease in the G0/G1 phase and a remarkable increase in the S phase. In contrast, the opposite effect was observed for A9D. The percentage of A9D cells in the S phase was similar to what is shown in Supplementary Figure S1 as expected; however, the number of A9D cells in the S phase was statistically lower than that found for S9D (Figure 4A), in which a 15% increase was observed. Similarly, the proliferation rate was evaluated through the expression of the Ki-67 marker (Figure 4B), showing, as expected, a higher expression of Ki-67 in A9D compared to S9D (p < 0.01), which is associated with a higher rate of proliferation.

Our results clearly show that a cell population enriched in CSCs (S9D) releases significantly more DNA into the extracellular medium than adherent cells (A9D) and that the profile of this DNA varies depending on its origin (i.e., whether it comes from spheres or adherent cells).

### 3.4 cfDNA from CSC-enriched cultures can be acquired passively and induce *in vitro* cell transformation

**FIGURE 5:**
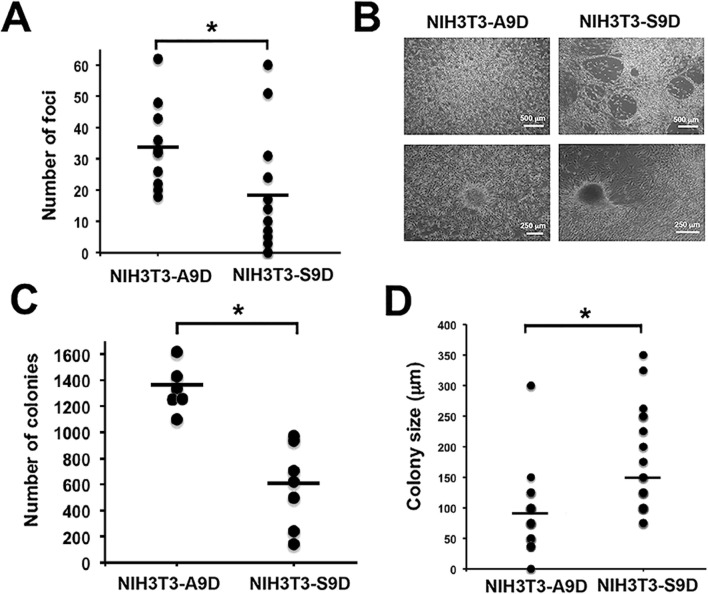
The cfDNA derived from A9D and S9D cells has a transforming capacity. The transforming capacity of the cfDNA was determined by *in vitro* experiments, showing the presence of acquired characteristics typical of a transformed cell: **(A)** Number of foci (evaluating loss of inhibition by contact) derived from NIH3T3 cells exposed to A9D and S9D supernatants (NIH3T3-A9D and NIH3T3-S9D). **(B)** Morphological differences under microscopic analysis. **(C)** Several colonies grown in soft agar (anchorage-independent growth assessment) derived from NIH3T3-A9D and NIH3T3-S9D foci. **(D)** Size of NIH3T3-A9D and NIH3T3-S9D colonies obtained in soft agar. ^*^p < 0.05.

It is recognized that CSCs have a high transforming, tumorigenic, and invasive capacity (Yin et al., 2021). Therefore, the next aim of the present study was to determine the *in vitro* transforming potential of the cfDNA released by S9D compared to that released by A9D. To achieve this objective, loss of contact inhibition (foci formation) and anchorage-independent growth (colony formation in soft agar) were assessed in the murine fibroblast NIH3T3 cell line, which was cultured with the supernatants of A9D (NIH3T3-A9D) or S9D (NIH3T3-S9D) for 3 weeks. During this period, passive transfer of cfDNA to this cell line has already been demonstrated (Trejo-Becerril et al., 2012). Foci counts were performed daily over a 3-week period (Figure 5A; p < 0.05), revealing a higher number of foci in NIH3T3-A9D cells compared to NIH3T3-S9D cells.

Interestingly, the foci found in NIH3T3-S9D differed morphologically from those of NIH3T3-A9D. The foci derived from NIH3T3-S9D were more rugged, exhibited fenestrated structures, and had a very compact consistency, as shown in Figure 5B. In contrast, NIH3T3-A9D cells exhibited homogeneous cell growth, and the foci they formed were of lax or not very dense consistency (Figure 5B).

To assess anchorage-independent growth, some foci from the previous assay were removed from the dish and seeded onto soft agar, where they were grown for 6 weeks. At the end of the 6 weeks, colonies were counted. We found that the foci obtained from the NIH3T3-A9D culture generated more colonies than those obtained in the NIH3T3-S9D culture, which was statistically significant (Figure 5C; p < 0.01). In addition to the difference in the number of colonies formed, we found that the colonies from the NIH3T3-S9D foci were larger compared to those generated from NIH3T3-A9D, as shown in Figure 5D, which was also statistically significant (p < 0.01). Furthermore, the colonies had distinct morphological characteristics. Colonies formed from the NIH3T3-A9D foci exhibited a less dense consistency, allowing individual cells to be discerned (Supplementary Figure S4A, upper panel). In contrast, colonies formed from the NIH3T3-S9D foci exhibited a very compact and dense consistency (Supplementary Figure S4A, lower panel).

Finally, to confirm that the colonies grown in soft agar originated from NIH3T3 cells transformed by cfDNA released from A9D or S9D cells (of human origin), genomic DNA was extracted from several colonies obtained in soft agar. A fragment of the human *KRAS* gene, including the mutated codon 12 (one of the most well-characterized pathogenic mutations in the SW480 cell line) and the *ALU* sequence specific to humans, was amplified using PCR. As shown in Supplementary Figures S4B, C, some of the colonies tested positive for the amplification of *KRAS* and *ALU* Yd6, respectively, thereby confirming the transfer of DNA from the supernatant of A9D and S9D cells to the NIH3T3 cells (of murine origin).

## 4 Discussion

Our study showed a clear correlation between the percentage of CSCs present in each cell line and the amount of cfDNA released, along with a characteristic electrophoretic profile for each cell line. Interestingly, we observed different transforming potentials depending on the origin of the cfDNA, which was related to the number of CSCs.

We employed three colon cancer cell lines (HCT116, HT29, and SW480), each exhibiting varying proportions of CSCs (CD133+/CD44+). For the first time in a colon cancer cell model, we demonstrated a direct relationship between the proportion of CSCs and the quantity of cfDNA released into the extracellular medium.

Our findings revealed that the HT29 cell line, despite not representing the most advanced clinical stage (Dukes’ C) among the analyzed cell lines (Ahmed et al., 2013), displayed the highest proportion of CSCs, as previously reported (Olejniczak et al., 2018; Wang et al., 2012), and released the greatest levels of cfDNA. Furthermore, we discovered that histological origin and cell proliferation rate are not determining factors for cfDNA release, which was unexpected given that earlier studies have indicated a direct correlation between cfDNA levels, tumor burden, and advanced stages of tumor progression (Barefoot et al., 2021).

Notably, the release of cfDNA was not linked to cell death or DNA replication processes. This observation is consistent with the findings of Wang et al. (2017), who reported that cfDNA concentrations do not correlate with the number of apoptotic or necrotic cells. Moreover, across all experiments, high cell viability was consistently observed, with most cells in the G0/G1 phase characterized by a 2C DNA content (de Miranda et al., 2021). This effectively rules out the possibility that elevated cfDNA levels are attributable to increased DNA content.

To confirm the direct correlation between the proportion of CSCs and the amount of cfDNA released into the extracellular medium, the CSC population in the SW480 cell line, which has the lowest proportion of CSCs (1.45% ± 0.375 CSCs), was enriched. This was accomplished using a modified non-adherent culture system (Chen et al., 2012), resulting in a significant increase in the proportion of CSCs (CD133+/CD44+) after 9 days of culture (S9D), achieving a 6.8-fold increase (9.87% ± 0.015) and an associated rise in the amount of cfDNA released.

Notably, the non-adherent culture system fosters the acquisition of stem-like characteristics, such as self-renewal and survival, along with the expression of Nanog, Sox-2, Oct-4, and c-Myc (Stankevicius et al., 2017). It also induces the surface markers CD133 and CD44, widely used to identify stem cells in colon cancer (Dotse and Bian, 2016; Olejniczak et al., 2018). Furthermore, these markers play key roles in anoikis resistance and cellular reprogramming through the interaction of CD44 with STAT3, which promotes the expression of Sox-2 and Oct-3/4 factors (Pan et al., 2017). Meanwhile, CD133+, whose expression can vary throughout the cell cycle or in response to different cellular conditions (Moreno-Londoño and Robles-Flores, 2024; Okada et al., 2021), is associated with signaling pathways such as PI3K/Akt and Wnt/β-catenin, which drive migration, invasion, metastasis, and proliferation, respectively (Glumac and LeBeau, 2018). Moreover, the detection of CD133+ and CD44^+^ in patient serum has been correlated with relapses and metastases in colorectal cancer, linked to the presence of latent CSCs (Wahab et al., 2017).

To verify the increase in the CSC population in the S9D cultures, we assessed the expression of the transcription factors Sox-2, Nanog, and Oct-3/4. The results showed a marked increase in their expression compared to the A9D cultures, indicating a higher stem cell content in the S9D cultures. Previous studies have reported that elevated Sox-2 expression is associated with reduced cell proliferation, a less adherent phenotype, and more spheroidal growth (Lundberg et al., 2016), aligning with our observations in these cultures.

Simultaneously, markers of cellular differentiation were evaluated, as these are influenced by the hypoxic conditions generated in non-adherent cultures (Okada et al., 2021). It was observed that E-cadherin expression was high in S9D cells. At the same time, N-cadherin was not expressed in the non-adherent cultures, and no significant differences in Vimentin expression were detected. Although these findings may initially appear contradictory, prior research has identified an interconnection between E-cadherin and Nanog expression, which promotes CSC self-renewal through interactions with Rap1 and other regulatory pathways, such as Wnt signaling (Tamura et al., 2018). This highlights the diversity within the CSC population at different stages of differentiation (Collura et al., 2013; Pan et al., 2017; Su et al., 2021).

Under our experimental conditions, the CSC population significantly contributes to the amount of cfDNA released—an important finding not previously reported. The observed increase in cfDNA levels by S9D cells could be attributed to the intrinsic characteristics of CSCs, which exhibit distinct cellular behavior influenced by their genomic context (Matikas et al., 2016). Alternatively, it may be linked to the hypoxic conditions of non-adherent 3D cultures, where low oxygen concentrations have been strongly associated with cfDNA release mechanisms and levels (Modi et al., 2021; Otandault et al., 2020). Additionally, molecular structures associated with cfDNA, such as extracellular vesicles (EVs), virtosomes, and nucleosomes, among others, may play a role (Gahan and Stroun, 2010; Umwali et al., 2021). These structures can be secreted as a cellular response to exogenous stimuli, such as hypoxia and acidification, to mitigate cellular stress (Li et al., 2022; Rostami et al., 2020).

In addition to increased cfDNA release, S9D cells exhibited a distinctive fragmentation pattern in the released cfDNA, comprising both high- and low-molecular-weight fractions, whereas A9D cultures displayed a broader size distribution. This fragmentation pattern is similar to what has been observed in the serum of cancer patients (Bi et al., 2020; Liu X et al., 2022), where it has been correlated with diagnostic and prognostic outcomes (Ding and Lo, 2022; Thierry, 2023). Furthermore, variations in cfDNA sizes have been linked to their cellular origin and the mechanisms of release, whether passive (cell death) or active secretion (Aucamp et al., 2018; Bronkhorst et al., 2022; Sanchez et al., 2021).

Regarding the latter, Stroun et al. demonstrated the spontaneous release of newly synthesised DNA in cell cultures (Stroun et al., 2001). It has also been proposed that large chromosomal regions, such as micronuclei or extrachromosomal circular DNA (eccDNA), can be released and transported via extracellular vesicles (EVs) (Alekseeva and Mironova, 2021; Bronkhorst et al., 2022; Bronkhorst et al., 2016; Morozkin et al., 2009; Morozkin et al., 2004; Tutanov and Tamkovich, 2022). Alternatively, they may be exported through various pumps or transporters, such as ABC transporters, which are abundantly expressed in CSCs (Begicevic and Falasca, 2017; Hatano et al., 2017). This mechanism could explain the presence of high-molecular-weight molecules in the cfDNA released by S9D cultures.

The low-molecular-weight cfDNA fraction (∼30–80 bp, with peaks at 42–60 bp) may correspond to mt-cfDNA (mitochondrial cell-free DNA) or sequences associated with enhancers and transcription factor binding sites in promoters (de Miranda et al., 2021; Sanchez et al., 2021; Zukowski et al., 2020). To achieve a more mechanistic understanding of the cellular processes in CSCs involved in cfDNA secretion, experimental strategies should be employed to inhibit relevant signalling pathways and cellular trafficking mechanisms.

Additionally, the fragment patterns and quantities of cfDNA released in each culture could be attributed to population diversity, with 3D cultures exhibiting greater heterogeneity than 2D cultures (Aucamp et al., 2017b). Alternatively, these differences might be linked to changes in the epigenetic environment of tumor cells, which affect DNA compaction and accessibility to DNases (Liu Y et al., 2019). Such changes could produce fragments of varying lengths with epigenetic modifications, such as methylation, that confer increased stability, resistance to enzymatic degradation (Cirmena et al., 2021), and specific cellular identity (Koval et al., 2021; Styk et al., 2022). However, in this study, no differences in global methylation levels were observed between A9D and S9D cultures, ruling out epigenetic regulation as the cause of the differences in cfDNA fragmentation.

The data obtained in this study demonstrate low expression of the proliferation marker Ki-67 in S9D cultures, indicating that the increase in cfDNA is not related to proliferative capacity, contrary to previous reports (Chen Z et al., 2005; Garcia-Arranz et al., 2017). However, cell cycle analysis revealed that the majority of cells in both A9D and S9D cultures were in the G0/G1 phase, consistent with findings by Wang et al., which suggest that cfDNA primarily originates from cells in the G1 phase (Wang et al., 2017). These cells are typically in a more differentiated state (Zaveri and Dhawan, 2018). Interestingly, a significant proportion of S9D cells were found in the S phase of the cell cycle, aligning with observations in pluripotent and embryonic stem cells (Baghdadchi, 2013; Symonds et al., 2009). Notably, during the S phase, the amount of genomic DNA increases, which could account for the elevated levels of cfDNA released by S9D cultures.

Previously, cfDNA was regarded as an inert byproduct released into the extracellular environment. However, its involvement in various biological functions has since been reported (Fernández-Domínguez et al., 2021), notably its ability to be horizontally transferred to recipient cells (Alekseeva and Mironova, 2021; Garcia-Arranz et al., 2017; To and Cho, 2022; Trejo-Becerril et al., 2012; Valcz et al., 2022). This transfer may carry pre-existing genetic alterations, potentially promoting the malignant transformation of recipient cells and contributing to tumor progression (Abdouh et al., 2019). It has been reported that cfDNA can activate TLR9 signaling, for instance, promoting autophagy and tumor growth. Additionally, it induces the overexpression of pro-metastatic genes such as MMP9, CD44, and CXCL8, and alters key pathways like TLR9/MYD88, facilitating cellular invasion and migration. On the other hand, it has been observed that cfDNA enriched in mobile retrotransposons, such as active LINE-1 elements, can integrate into the genomes of recipient cells through “cut and paste” mechanisms, inactivating tumor suppressor genes and promoting the formation of new tumors (Niu et al., 2018; Grabuschnig et al., 2020). And recently, Filatova AA et al. (2023) demonstrated that cfDNA from mice with melanoma is capable of penetrating B16 cells, leading to an increase in the abundance of oncogenes and fragments of mobile genetic elements (MGE). This phenomenon causes an increase in the mRNA levels of Dnase 1L3 and in the mRNA levels of the oncogenes Jun, Fos, Ras, and Myc. These oncogenes, in turn, trigger various signaling cascades, ranging from the inhibition of apoptosis to the increased proliferation of tumor cells.

*In vitro*, assays such as focus formation and anchorage-independent growth are commonly employed to assess malignant transformation (Chatterjee and Alfaro-Moreno, 2023). In this study, the NIH3T3 cell line was used as recipient cells. Our results revealed a greater number of foci in NIH3T3 cells exposed to the A9D culture supernatant (NIH3T3-A9D) compared to those exposed to the S9D culture supernatant (NIH3T3-S9D), despite the latter containing higher levels of cfDNA. These data suggest that the disparity in effect could largely be attributed to the nature of the cfDNA released by each cell clone. In the case of A9D, the cfDNA populations released into the conditioned medium consist of fragments of varying sizes (Figures 3C–E). In contrast, the cfDNA released by S9D cells exhibits two well-defined populations: one of high molecular weight and another of very low molecular weight (Figures 3C, D, F). Given that the transfer of complete oncogenes could explain the transformation caused by high molecular weight cfDNA, the observation that S9D transforms fewer cells but with greater efficiency suggests that this effect is precisely due to the increased release of high molecular weight cfDNA.

Morphological differences were also observed in the foci formed. NIH3T3-A9D cells displayed type III invasive growth with multiple cell layers, whereas NIH3T3-S9D cells formed denser, more compact foci, consistent with descriptions in the literature (Sasaki et al., 2012). Anchorage-independent growth was evaluated by colony formation in soft agar, revealing that colonies derived from NIH3T3-S9D cells were fewer but larger than those from NIH3T3-A9D cells, indicating a higher rate of cell proliferation (Mascolo et al., 2018). Additionally, colonies derived from NIH3T3-A9D cells were heterogeneous with irregular edges, while those from NIH3T3-S9D cells displayed a compact morphology with well-defined edges. These findings demonstrate that cfDNA from the A9D and S9D culture supernatants possesses transforming potential in NIH3T3 cells. The distinct characteristics of the colonies suggest that their morphology and behavior are influenced by the origin of the cfDNA to which the cells were exposed. Several studies indicate that not all cfDNA sequences can transform cells unless they originate from neoplastic cells. For example, Tuomela et al. (2013) demonstrated that cfDNA released from MDA-MB-231 cells induced to undergo cell death by doxorubicin can promote the invasion of this same cell line in its wild-type form when exposed to cfDNA derived from cellular debris. In contrast, Garcia-Arranz et al. (2017) showed that cfDNA derived from non-neoplastic cells inhibits cell division, reduces tumor size, and partially blocks metastasis. Meanwhile, Mittra I et al. (2015) reported that cfDNA isolated from the blood of healthy volunteers can damage the DNA of healthy cells by integrating into their genomes, ultimately causing cell death.

The positive amplification of the *KRAS* gene and the human repetitive sequence *ALU* Yd6 in NIH3T3-A9D and NIH3T3-S9D cells confirmed the transfer of human cfDNA to murine cells. *KRAS* was selected due to its frequent colorectal cancer mutation, prevalence in patient plasma, and established role in cellular transformation (Alekseeva and Mironova, 2021; Xie and Kim, 2021). Meanwhile, the *ALU* sequence was utilized to verify the transfer of human genetic material to the murine clones NIH3T3-A9D and NIH3T3-S9D (Walker et al., 2003).

The findings of this study suggest a critical association between CSCs and the quantity of cfDNA released. Furthermore, cfDNA displays a distinctive fragmentation profile and can transfer malignant characteristics to recipient cells, promoting cellular transformation. CSCs are pivotal in tumor heterogeneity, initiation, metastasis, therapeutic resistance, and recurrence, exhibiting a remarkable ability to adapt to diverse tissue microenvironments during neoplastic invasion. As such, a comprehensive analysis of cfDNA may provide essential insights into the biology and behavior of CSCs.

It is important to emphasize that our study focused exclusively on 3 cell lines of the same cancer type, which may limit the generalizability of the results to other cell lines or neoplasms. Nevertheless, the findings are consistent and reproducible, highlighting the need to expand the analysis to additional cell lines from various cancer types and non-cancerous cell cultures. Given the significance of these findings, tumorigenicity assays in murine models are essential to investigate further the transformative capacity of cfDNA released by CSCs. It is also important to note that the cfDNA released by the isolated CSCs originates from the total supernatant extract, which contains all potential molecular structures or complexes cfDNA may associate with. Therefore, it is imperative to conduct future assays to determine the predominant structural form of cfDNA present in the S9D culture.

Likewise, assessing the clinical relevance of these results in colon cancer patients is essential. This includes establishing the relationship between cfDNA concentrations in plasma or serum, the proportion of CSCs in the primary tumor, and the clinical progression of the disease.

Finally, the results of our study have generated valuable and insightful data that can inform future experimental approaches. Although our findings suggest a critical association between CSCs and the amount of cfDNA released in the *in vitro* colon cancer model, the underlying mechanism driving this phenomenon remains unknown.

## 5 Conclusion

Our study demonstrated for the first time that there is a relationship between the proportion of CSCs in cell culture and the amount of cfDNA released into the extracellular medium. This was proven by enriching the stem population in a cell line with a low percentage of CSCs.

Under our experimental conditions, we determined that the electrophoretic profile of cfDNA derived from an adherent monolayer culture (2D) differs considerably from the profile of cfDNA derived from a sphere culture (3D) of the same cell line (SW480). Additionally, we showed that cfDNA can enter a recipient cell and induce transformation, which has already been demonstrated. Interestingly, we also showed that depending on the cellular origin of the cfDNA, different transformation characteristics were observed in NIH3T3 cells exposed to the different supernatants.

CSCs play a crucial role in therapy resistance and cancer recurrence, while cfDNA has established itself as a valuable molecular marker in the diagnosis, treatment, and monitoring of cancer. It reflects the epigenetic and genetic characteristics of the cells from which it originates. Therefore, CSCs may transfer resistance mutations to surrounding cells through cfDNA, which may maintain disease regionally or keep metastases dormant. Consequently, longitudinal sampling of cfDNA would enable the identification of acquired resistance mutations and the tracking of the tumor’s clonal evolution. This would facilitate the detection of minimal residual disease and monitor the patient’s response to various therapies. This information could significantly contribute to developing more effective treatments and a better selection of therapeutic regimens.

In conclusion, our results provide crucial information that could accelerate the translation of cfDNA analysis into clinical practice and help elucidate the nature of cfDNA itself.

## Data Availability

The original contributions presented in the study are included in the article/Supplementary Material, further inquiries can be directed to the corresponding authors.

## Supplementary material

SUPPLEMENTARY FIGURE S1 The amount of cfDNA released by colon cancer cell lines is independent of cell cycle phases. To determine the influence of the cell cycle on DNA release, cell cycle analysis was performed by flow cytometry in the three colon cancer cell lines. Statistically significant differences were found in the S and G2/M phases; however, this was unrelated to the amounts of cfDNA released by each line. ^*^p < 0.05, ^**^p < 0.01.

SUPPLEMENTARY FIGURE S2 Characterization of the SW480 cell line enriched in CSCs. Enrichment was carried out for 9 days using a non-adherent culture that favors the formation of spheres **(A)** Morphological features of spheres from SW480 cells (S9D). Heterogeneous spherical structures without dense packing and irregular external edges, with individual cells favoring a loose structure, are observed **(B–D)**. Expression of markers is associated with cellular differentiation. Significant differences were only found in the expression of E-cadherin concerning the other proteins evaluated. Interestingly, we did not find N-cadherin expression at any time evaluated (3, 5, and 9 days) in both adherent cells and SW480 spheres, however, it was highly expressed in the positive control. ^*^p = 0.0007. There was no expression of E-cadherin in the positive control due to the cadherin switching that occurs during EMT, where high N-cadherin expression substitutes for E-cadherin expression (Loh et al., 2019).

SUPPLEMENTARY FIGURE S3 The size of cfDNA fragments released to the extracellular medium is unrelated to methylation levels. Genomic DNA and cfDNA from A9D and S9D cells were subjected to global methylation assay. There was no difference between the methylation levels of the cfDNA obtained from the supernatants of A9D and S9D, which is similar to what was observed in the genomic DNA of the same cells. cfDNA = cell-free DNA; DNA = genomic DNA.

SUPPLEMENTARY FIGURE S4 Morphological and molecular characterization of NIH3T3 cells transformed by cfDNA. **(A)** Microphotograph of colonies grown on soft agar. Colonies derived from NIH3T3/A9D cells are irregular compared to those obtained from NIH3T3/S9D, which show a more compact and dense morphology. **(B)** Amplification of a fragment of the human *K-ras* gene that included the mutated codon 12. Two clones obtained from NIH3T3/A9D and two of NIH3T3/S9D were positive for *K-ras* amplification, confirming the acquisition of cfDNA in the supernatants. **(C)** Amplification of *ALU* sequence specific to humans. Two clones obtained from NIH3T3/A9D and two of NIH3T3/S9D were positive for *ALU* amplification, confirming the acquisition of cfDNA in the supernatants.

## Abbreviations

FBS, fetal bovine serum; PBS, phosphate-buffered saline; PCR, polymerase chain reaction; bp, base pair; cfDNA, cell-free DNA; mt-cfDNA, mitochondrial cfDNA; EVs, extracellular vesicles; CSC, cancer stem cells; CCSCs, colon cancer stem cells; A-SW480, adherent SW480 cells; S-SW480, spheres SW480 cells; A9D, adherent culture of 9 days; S9D, spheres (3D) culture of 9 days; NIH3T3-A9D, NIH3T3 cells exposed to supernatant derived from A9D cells; NIH3T3-A9D, NIH3T3 cells exposed to supernatant derived from S9D cells; CT+, positive control; DNA, DNA genomic; PAGE, Polyacrylamide gel electrophoresis.
