# Supplementary figures and images for: Increased amounts of cell-free DNA released from a culture with a high content of cancer stem cells
Source: Front Cell Dev Biol. 2025 Mar 28;13:1499936. doi: 10.3389/fcell.2025.1499936 (PMC11985834; doi:10.3389/fcell.2025.1499936)

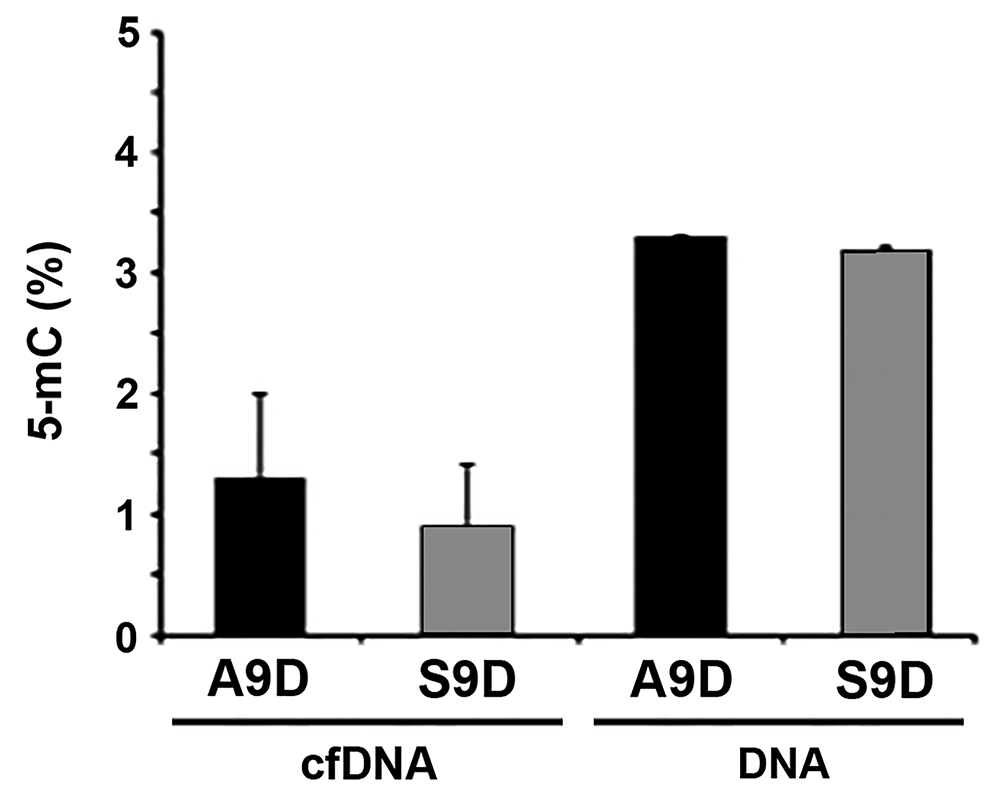

Supplement: Supplementary file 1 [file Image3.tif]

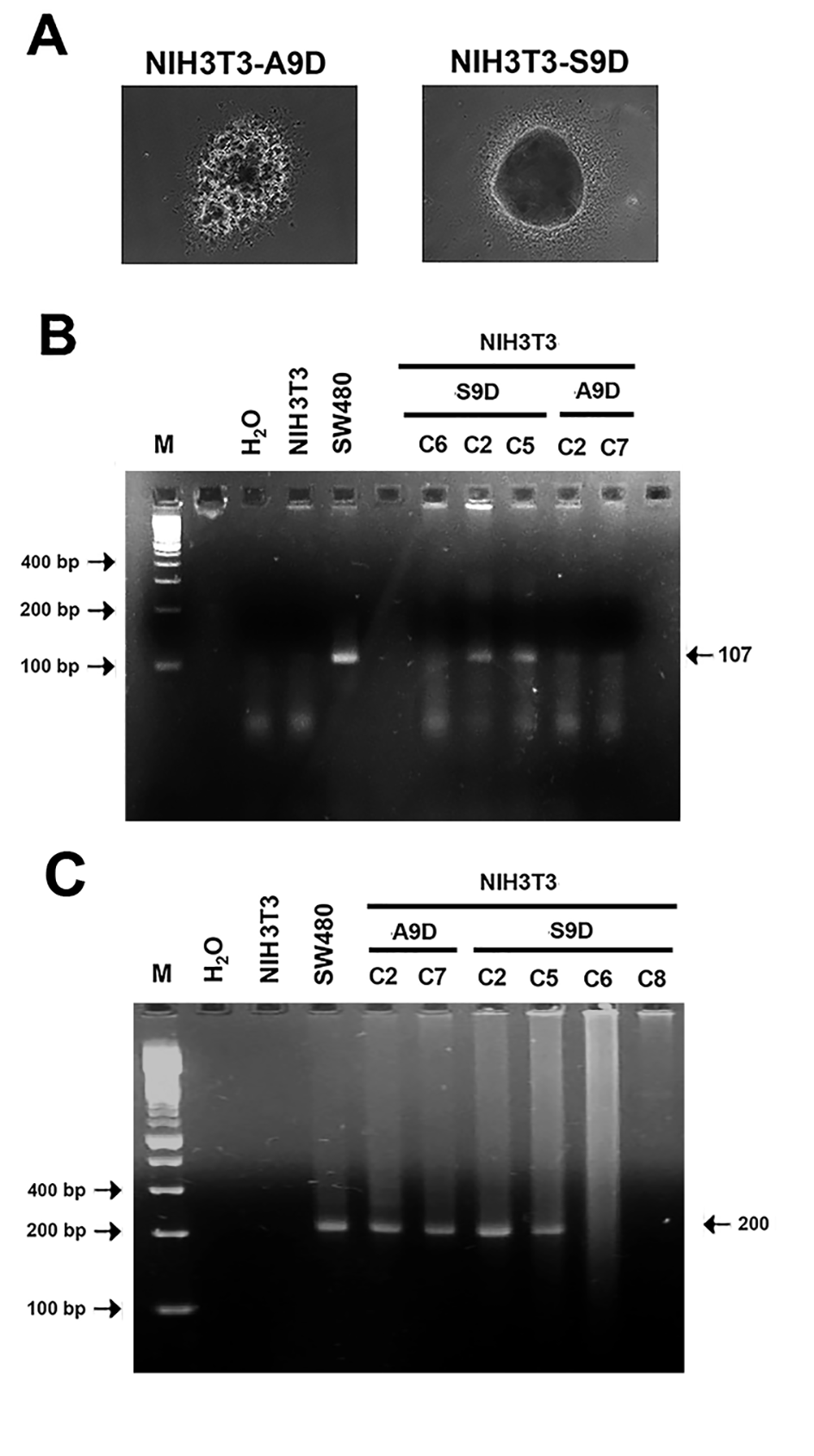

Supplement: Supplementary file 2 [file Image4.tif]

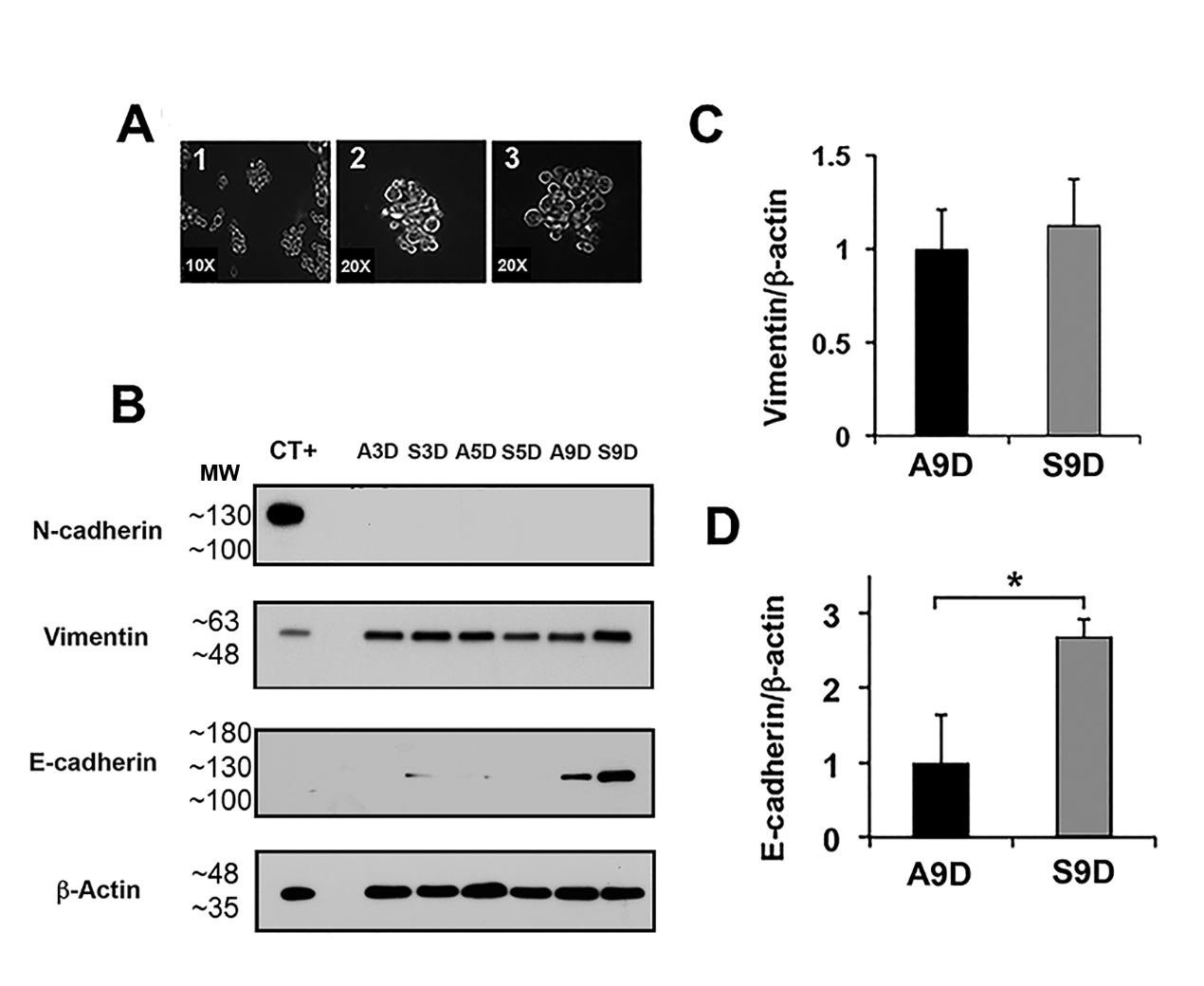

Supplement: Supplementary file 3 [file Image2.tif]

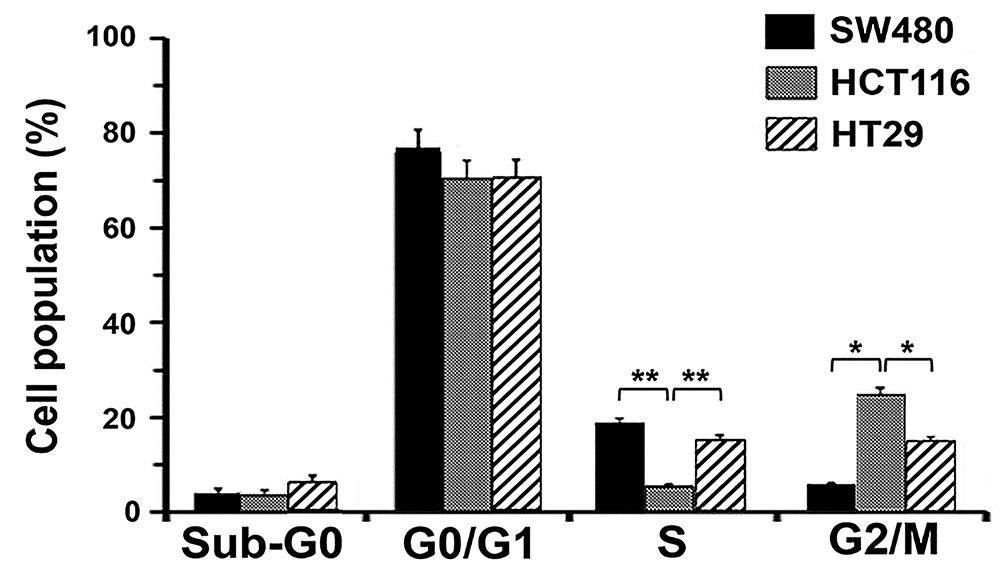

Supplement: Supplementary file 4 [file Image1.tif]
